# Low-Income US Women Under-informed of the Specific Health Benefits of Consuming Beans

**DOI:** 10.1371/journal.pone.0147592

**Published:** 2016-01-28

**Authors:** Donna M. Winham, Traci L. Armstrong Florian, Sharon V. Thompson

**Affiliations:** 1 Food Science & Human Nutrition, Iowa State University, Ames, Iowa, United States of America; 2 Cooperative Extension Maricopa County, University of Arizona, Phoenix, Arizona, United States of America; 3 Food Science & Human Nutrition, University of Illinois at Urbana-Champaign, Urbana, Illinois, United States of America; University of Louisville, UNITED STATES

## Abstract

**Background:**

Bean consumption can reduce chronic disease risk and improve nutrition status. Consumer knowledge of bean health benefits could lead to increased intakes. Low-income women have poorer health and nutrition, but their level of knowledge about bean health benefits is unknown. Beans are a familiar food of reasonable cost in most settings and are cultural staples for Hispanics and other ethnicities. Study objectives were to assess awareness of bean health benefits among low-income women, and to evaluate any differences by acculturation status for Hispanic women in the Southwestern United States.

**Methods:**

A convenience sample of 406 primarily Mexican-origin (70%) low-income women completed a survey on knowledge of bean health benefits and general food behaviors. Principal components analysis of responses identified two summary scale constructs representing “bean health benefits” and “food behaviors.” Acculturation level was the main independent variable in chi-square or ANOVA.

**Results:**

The survey completion rate was 86% (406/471). Most women agreed or strongly agreed that beans improved nutrition (65%) and were satiating (62%). Over 50% answered ‘neutral’ to statements that beans could lower LDL cholesterol (52%), control blood glucose (56%) or reduce cancer risk (56%), indicating indifference or possible lack of knowledge about bean health benefits. There were significant differences by acculturation for beliefs that beans aid weight loss and intestinal health. Scores on the bean health benefits scale, but not the food behavior scale, also differed by acculturation.

**Conclusions:**

Limited resource women have a favorable view of the nutrition value of beans, but the majority did not agree or disagreed with statements about bean health benefits. Greater efforts to educate low-income women about bean health benefits may increase consumption and improve nutrition.

## Introduction

Dried beans of the *Phaseolus vulgaris L*. species such as pinto, black, kidney, or navy are high in protein, fiber, folate, iron, magnesium, and other micronutrients essential for optimal health. Regular bean consumption as a part of a healthy daily diet is associated with increased longevity [1], reduced cardiovascular disease risk [2–4], and reduced reoccurrence of colorectal polyps [5]. For persons with type 2 diabetes, bean consumption can improve blood glucose control [6]. In fact, beans gained prominence in the 2005 and 2010 Dietary Guidelines for Americans (DGA) by being clearly placed in the vegetable as well as protein categories of MyPyramid and MyPlate [7,8]. In recent years, the United States Department of Agriculture (USDA) has promoted the nutrition and health benefits of dried beans across multiple nutrition assistance programs. Retention or inclusion of dried beans in the diets of limited resource populations is one possible way to increase vegetable intake, improve dietary quality, and decrease chronic disease risk [9,10]. In 2010, beans were incorporated into the Special Supplemental Food Program for Women, Infants and Children (WIC) food package to not only improve nutrition options, but also to offer culturally sensitive alternatives for immigrants and minorities [11].

The health-promoting attributes of food patterns high in beans, such as the Mediterranean diet, have been known for over 30 years [9,12]. More recently, a four-country study by Darmadi-Blackberry et al. found that for every 20 gram increase in legume consumption risk of death among the older adults decreased by 8% [1]. Considering that one-half cup of cooked beans weighs about 100 grams depending on type, 20 grams is a small amount to consume [13]. Results from the US National Health and Nutrition Examination Survey I Epidemiologic Follow-up Study indicated that legume consumption four or more times per week reduced heart disease risk by 22% in contrast to intakes of less than once a week [2]. A 7–9% reduction in LDL and total cholesterol has been observed in two studies that examined the effect of consuming one-half cup pinto beans daily over an 8–12 week period [14,15]. Similar cholesterol reduction effects across a wider range of *P*. *vulgaris* species were supported by meta-analysis [16]. Beans decrease postprandial hyperglycemia alone or as part of higher glycemic index meals, thus potentially reducing damage from elevated blood glucose [6,17]. Bean consumption is also linked to a reduction in polyps, which are associated with heightened colon cancer risk [5].

Despite these many health benefits, dried bean consumption has been declining in the US, Europe, and many other countries around the world [10,18]. Westernization of the diet, increased purchasing power, and acculturation are several forces that contribute to declines in bean intakes [18,19]. In the US, about 8% of the total population consumes beans each day, but among Hispanics, this percentage may be as high as 25% [18]. However, more frequent daily consumption does not mean Hispanic consumers meet the DGA recommended amounts. An earlier study with low-income women in Phoenix, Arizona, USA, showed that Hispanic women ate more beans than their non-Hispanic peers, but neither group met the DGA recommended intakes [19].

Populations with low incomes in the US experience a disproportionate chronic disease burden, in particular cardiovascular disease (CVD), diabetes, and obesity, than those with higher earnings [20,21]. Acculturation issues and language barriers can worsen the health of immigrants and minority groups with low incomes even further. Some immigrants to the US adopt negative dietary habits in their new surroundings as part of acculturation, time constraints on food preparation, and accessibility of foods [20,21]. Dietary changes towards a more Westernized diet may already have happened in the country of origin as well [10,19,22]. Increased consumption of high saturated fat items such as red meats and fast foods, larger portions of processed foods, and decreased intakes of traditional foods like beans can elevate chronic disease risk [10,22–24]. The pattern of dietary acculturation is dependent upon many sociocultural factors at the individual and group level and varies by ethnicity as well as geographically [22–24]. Meeting nutrition recommendations for groups with limited resources may be difficult in the face of poverty, cultural expectations, and restricted time [20,25,26]. Thus, retention of traditional dietary patterns that include beans may be a logical and culturally acceptable way to improve nutrition and health [23,27].

Survey research data were collected from predominately Hispanic, limited resource women in metropolitan Phoenix, Arizona, USA. The study objectives were to 1) assess knowledge of the health benefits of beans among women with limited resources who were eligible for nutrition programs such as WIC, Supplemental Nutrition Assistance Program (SNAP), and the Expanded Food Nutrition Education Program (EFNEP), and 2) identify predictors of knowledge such as education or acculturation status. We hypothesized that participants’ nutrition knowledge of the health benefits of beans would be lower among less acculturated participants.

## Methods

### Study Design and Participants

A convenience sample of low income women aged 18–65 years who were currently enrolled or eligible to participate in US federal nutrition programs in metropolitan Phoenix, Arizona completed the self-administered survey. In general, adults with a household income less than 185% of the federal poverty guidelines, based on their household size, are eligible to participate in most programs [28,29]. Recruitment was conducted at three different venues between May-December 2011 including regular EFNEP classes (12 days), a WIC clinic (5 days), and an unemployment job center (3 days). Research permissions were obtained from site directors: TL Armstrong Florian, Maricopa County EFNEP, K. Sell, Maricopa County WIC, and T. Ferrell, Maricopa County Workforce job center. Sites were chosen based on the high presence of Hispanic clientele as determined from program and center access reports. Data were collected from all interested and eligible women, including non-Hispanics, at the study venues.

### Bean health benefit knowledge survey development

An ethnographic interview guide was developed for use in focus groups with three EFNEP classes (n = 31), and one-on-one interviews with four different nutrition assistance program staff (3 EFNEP; 1 WIC). In-depth qualitative information focusing on beans in traditional diet changes, cultural preferences for bean characteristics, cultural acceptability, and social marketing of beans guided the development of a structured questionnaire. These cognitive interviews shaped recruitment strategies, question wording, and identified potential barriers and motivators to participation before implementation. Eight public health nutrition researchers reviewed the final survey instrument for content validity.

Seven 5-point Likert-type questions about the purported health benefits of beans were pilot tested in English with 14 nutrition graduate students and again with 4 laypersons for wording and clarity. Two different bilingual Mexican-American researchers translated the instrument into Spanish and back-translated it to English. The EFNEP field staff and researchers reviewed the Spanish version and developed consensus on the appropriate colloquial phrasing for limited resource Mexican-origin women. The final Spanish questionnaire was pilot tested again in three different EFNEP classes (n = 23). Minimal changes were made in formatting and wording based on pilot test feedback before official data collection began.

### Other survey instruments

Acculturation status was determined from the Bidimensional Acculturation Scale (BAS) [30]. The BAS assesses both English and Spanish use of media and social engagement through 24 questions that reflect cultural affinity in at least two dimensions (Hispanic, non-Hispanic). An average score between 0 and 5 is calculated for each domain. Values above 2.5 indicate higher affinity to one cultural domain or another. Persons scoring above 2.5 in both the Hispanic or non-Hispanic domain are classified as bicultural. Thus, the BAS goes beyond simple acculturation categorization by Spanish language use or self-reported Hispanic ethnicity as operationalized in the US Census. The standard national EFNEP agency enrollment form was utilized at all three venues for questions on demographics, ethnicity, race, household composition, income, money spent on food, and a 10-item food behavior checklist [31]. Instruments were used in their published English and Spanish versions.

### Survey Administration and Data Collection

For EFNEP participants, the surveys were self-administered during the third class session of the program’s six class series. The bilingual EFNEP instructional specialists were responsible for introducing the survey with at least one bilingual study researcher present to assist with data collection. EFNEP participants received incentives such as food coupons, pens, brand-marketed notepads, and shopping bags. At the WIC clinic and the Job Center, two to four study representatives set up a table, posted signs, and distributed flyers about the study to clients as they waited for services. Interested individuals selected questionnaires in English or Spanish dependent on their preference. An investigator read each participant a verbal script describing the study prior to the issuance of survey forms. Completion of the forms was considered assent and no personal identifying information was collected. The Arizona State University Institutional Review Board approved this study (IRB# 1009005462). All surveys were checked for completeness on site and any missing data were reviewed with the participant. Individuals who completed the survey at the WIC clinic or Job Center received $3.00 in cash.

### Data Analysis and Scale Construction

Data entry, transformations, and analyses were conducted using SPSS Statistics, Version 21.0 (IBM, Armonk, NY). Acculturation classifications of Hispanic dominant, Bicultural, or English dominant were computed in accordance with the published instructions for the BAS [30]. Relationships between Likert-type questions on the health benefits of beans and demographic, acculturation, household composition, and other variables were explored using chi-square analysis, correlations, and ANOVA as appropriate. After viewing the descriptive statistics for the bean health benefit knowledge questions and the 10-item food behavior checklist, principal components analysis (PCA) was used to examine clustering of Likert item questions to create scales for use in multivariate predictive modeling. The Kaiser-Meyer-Olkin measure confirmed sampling adequacy. Bartlett’s test of sphericity verified that item correlations were large enough for PCA, and eigenvalue and scree plots were examined to reveal two underlying constructs [32]. Likert score means were created by summing the variable scores and dividing by the total number of questions. The bean health benefits knowledge construct represented awareness of the health benefits of beans. The food shopping behaviors construct reflected a cluster of food safety and nutrition practices. The Cronbach’s alpha was 0.86 for the bean health benefits scale and 0.74 for the food shopper behavior scale [32]. Both scales were normally distributed.

## Results

A total of 471 women aged 18–65 years started the survey and 86% (n = 406) completed it. Most incomplete surveys were due to women at WIC leaving for their scheduled appointment midway through the survey. The majority of the participants were from WIC (46%), followed by EFNEP (36%), with a smaller percentage from the Job Center (18%). Data were pooled on the basis of eligibility for nutrition assistance programs from the three venues. However, there was a significant difference in mean age between the WIC (30 years ± 10.6 sd), EFNEP (35 years ± 8.0 sd), and the Job Center (43 years ± 12.0 sd) women. Survey responses indicated that the amount of money spent on food was similar between groups, but the Job Center women had fewer children and smaller household sizes.

Across study sites almost three-quarters of the women surveyed (71%) self-identified as Hispanic by the US Census ethnicity question and 62% self-identified as Mexican or Mexican-American on a separate demographic question. Only 36% of the 406 women were classified as Hispanic dominant (less acculturated) with 23% were categorized as bicultural, and 41% as English dominant by the BAS. Descriptive statistics were analyzed by both ethnicity (Hispanic; non-Hispanic) and the BAS acculturation scale categories (Hispanic dominant or less acculturated, Bicultural, English dominant or more acculturated). Both Hispanic and non-Hispanic women are included in the English dominant (more acculturated) grouping because the BAS reflects acculturation, and not simply self-reported Hispanic ethnicity. Descriptive statistics by BAS acculturation categories are shown in Table 1. Hispanic dominant women reported fewer years of education, more children, larger household size, lower household incomes, and were more likely to be in a married or cohabitating relationship than Bicultural or English dominant Hispanic or non-Hispanic women. Bicultural women were significantly younger than the other two groups by about 2–3 years. Despite differences in household size and composition by the BAS acculturation categories, the average amount of money spent on food each month was not significantly different.

**Table 1:** Distribution of demographic and household characteristics of women by Bidimensional Acculturation Scale classifications. (mean ± SD, or percentage) (n = 406).

Characteristics	Total	Hispanic dominant 36% (145)	Bicultural 24% (96)	English dominant 40% (165)
Age in years (±SD)*	34.1 ± 10.6	34.2 ± 7.3	31.8 ± 10.1	35.4 ± 13.0
Nativity ***				
Not born in the US	50.7	97.3	59.4	4.3
Born in the US	49.3	2.7	40.6	95.7
Self-Report as Hispanic				
Yes	71.0	100.0	96.9	70.0
No	29.0	0.0	3.1	30.0
Years of Education***				
6^th^ grade or less	9.9	26.2	2.1	0.0
7–9^th^ grade—Junior High	13.8	26.9	12.5	3.0
10–11^th^ grade	13.1	13.8	19.8	8.5
12^th^ grade or GED	22.7	22.1	22.9	23.2
Some college or tech school	25.2	6.9	25.0	41.5
Associates degree or more	15.3	4.1	17.7	23.8
Marital Status ***				
Single/Divorced	39.8	16.6	38.5	61.0
Married/Cohabitating	60.2	83.4	61.5	39.0
Number of children under age 20***	2.2 ± 1.4	2.7 ± 1.2	2.3 ± 1.3	1.7 ± 1.5
Number of adults***	2.2 ± 1.0	2.4 ± 1.1	2.5 ± 1.0	2.0 ± 0.9
Total household size***	4.4 ± 1.9	5.1 ± 1.6	4.8 ± 1.8	3.6 ± 1.9
Monthly amount spent on food $	286 ± 204	286 ± 187	290 ± 215	285 ± 213
Household monthly income $***	1429 ± 1234	1124 ± 698	1366 ± 1106	1748 ± 1575

*P < 0.05;

** P < 0.01;

*** P < 0.001.

For the food behavior checklist, a majority of respondents reported that they compared prices, shopped with a list, and thought about healthy food choices “most or all of the time” [31]. Only 35% indicated they used nutrition facts labels “most or all of the time.” Over 58% noted they thawed frozen foods at room temperature “sometimes to always.” Significant differences by acculturation status emerged for five of the ten individual questions. These were the same questions identified by PCA and used to create a summary score. No mean differences by acculturation category were observed in the food behavior Likert scale values.

Table 2 portrays the frequency of responses for the seven questions about the health benefits of beans. The five questions identified by PCA and used in the summary scale are noted. The majority of women agreed or strongly agreed that eating beans can improve your nutrition (66%) and help you feel full (62%). Conversely, over half reported neutral with regard to beans lowering ‘bad’ cholesterol (52%), lowering cancer risk (54%), or controlling blood sugar (56%). Women who were bicultural and English dominant (52–53%) were significantly more likely to agree or strongly agree that beans aided intestinal health than Hispanic dominant women (31%). More Hispanic dominant women (37%) disagreed or strongly disagreed that beans could help in weight loss whereas 26% of bicultural and 16% of English dominant women disagreed with this statement. The mean health benefits knowledge scale was 3.15 or midrange for all women, but with significantly higher values for English dominant, followed by those classified as Bicultural in comparison to Hispanic dominant women.

**Table 2:** Percentage of responses regarding health benefits of beans among low-income women by Bidimensional Acculturation Scale category (n = 406).

*Eating beans can …*.	Strongly Disagree	Disagree	Neutral	Agree	Strongly Agree
**1. Improve Your Nutrition**	**2.5**	**3.7**	**28.3**	**44.5**	**21.1**
Hispanic dominant	2.7	3.4	24.0	47.3	22.6
Bicultural	3.1	3.1	29.2	41.7	22.9
English dominant	1.8	4.2	31.5	43.6	18.8
**2. Help You Feel Full**	**4.4**	**6.9**	**26.8**	**44.2**	**17.7**
Hispanic dominant	7.5	8.9	26.0	43.8	13.7
Bicultural	3.1	9.4	24.0	47.9	15.6
English dominant	2.4	3.6	29.1	42.4	22.4
**3. Lower Bad Cholesterol**	**5.4**	**10.3**	**51.8**	**24.1**	**8.4**
Hispanic dominant	8.9	12.3	51.4	19.2	8.2
Bicultural	5.2	11.5	49.0	25.0	9.4
English dominant	2.4	7.9	53.9	27.9	7.9
**4. Lower Cancer Risk**	**6.4**	**9.6**	**53.6**	**23.1**	**7.4**
Hispanic dominant	8.9	15.1	51.4	19.2	5.5
Bicultural	6.3	5.2	57.3	22.9	8.3
English dominant	4.2	7.3	53.3	26.7	8.5
**5. Control Blood Sugar**	**5.7**	**12.0**	**56.3**	**20.6**	**5.4**
Hispanic dominant	9.6	15.1	53.1	17.8	4.1
Bicultural	5.2	13.5	53.1	20.8	7.3
English dominant	2.4	8.5	60.6	23.0	5.5
6. Healthy GI Tract***	**6.1**	**8.1**	**40.8**	**32.7**	**12.3**
Hispanic dominant	10.0	12.3	45.9	23.3	7.5
Bicultural	6.3	9.4	32.3	37.5	14.6
English dominant	1.8	3.6	41.2	38.2	15.2
7. Help Lose Weight***	**8.4**	**17.7**	**49.6**	**18.4**	**5.9**
Hispanic dominant	13.0	24.0	38.4	20.5	4.1
Bicultural	10.4	16.7	56.3	10.4	6.3
English dominant	3.0	12.7	55.8	21.2	7.3
**Summary scale**	**Total**	Hispanic dominant	Bicultural	English dominant	
Knowledge of health benefits of beans (±SD)*** (Sum of questions 1–5)	3.15 ± .75	2.95 ± .75	3.17 ± .81	3.32 ± .69	

*** P < 0.001.

A multivariate linear regression model was developed to determine if differences in the bean health benefits scale were predicted by ethnicity, acculturation level, nativity, demographic characteristics (age, education), or the food behavior scale. Table 3 shows the multivariate model. Nearly 14% of the variance in the bean health benefits knowledge score was predicted by a model including the food shopper behavior score, not being Hispanic dominant, and increasing age in years (F = 21.80, adjusted R^2^ = .135, p<0.000) [32].

**Table 3:** Significant predictors of bean health benefits score (n = 406).

Variable	B (SE)	Beta (p value)	F value	Adj R^2^	Model p
***Bean health benefits knowledge score***			21.93	.136	.000
Constant	1.98 (.185)				
Proactive shopper	.251 (.044)	.269 (.000)			
Hispanic dominant—BAS	-.295 (.073)	-.188 (.000)			
Age in years	.011 (.003)	.162 (.001)			

## Discussion

Limited resource populations including immigrants and Hispanics continue to have higher chronic disease risk and obesity in comparison to those with higher socioeconomic status. The relationship between these variables of disease risk, income, socioeconomic status, culture, and nutrition knowledge is complex, but is often mediated by acculturation [22,23]. Bean consumption has been linked with reduced disease risk in previous studies [1–6,9,16]. National consumption data as well as localized analysis of dietary patterns indicates bean consumption declines whereas chronic disease risk increases with greater acculturation by immigrants [10,18,23,24]. Thus knowledge of the health benefits of beans or other traditional foods that is based within cultural values may be a feasible approach to manifesting behavior change and reducing chronic disease risk, particularly for diabetes and cardiovascular disease for which diet has a strong etiological connection [19,27,33].

The US Centers for Disease Control estimate that 25.8 million people or 8.3% of the US population have diagnosed or undiagnosed type 2 diabetes. A disproportionate number of Hispanics (11.8%) have diagnosed diabetes, with this figure expected to rise [34]. Dietary changes and increased physical activity are the first line of treatment for persons with type 2 diabetes [35]. However, difficulty with adhering to dietary recommendations is one of the most frequently reported concerns among all persons with type 2 diabetes, and among Hispanics [36–38]. Two adherence barriers mentioned in other studies were specifically the exclusion of cultural foods and the inability to eat the same foods as the family [37,38]. In some cases, the food restrictions may be unwarranted. Jimenez-Cruz et al. found that traditional Mexican foods like corn tortillas and pinto beans had a low glycemic index, were satiating, and improved glycemic control in adults with type 2 diabetes [39]. Thompson et al. demonstrated that bean-and-rice meals blunted the glycemic response in comparison to a meal of rice alone matched on available carbohydrate among persons with type 2 diabetes [17]. With rates of diabetes increasing globally, indigenous and traditional foods, such as beans, may be the only treatment option in the absence of adequate and affordable medical care [23,27].

Cardiovascular disease (CVD) remains a serious health problem in the US. At least 100 million Americans have high cholesterol and are at increased risk of CVD, the leading cause of death in the US [40]. In a national survey sample of Mexican Americans, 44% of women and 48% of men had elevated cholesterol levels [40]. As with type 2 diabetes, dietary and lifestyle changes are recommended as the first intervention step to reduce CVD risk by improving blood lipid profiles [41]. Several studies have examined cholesterol reduction with portfolio diets as well as single food dietary interventions such as oatmeal, nuts, pomegranates, and beans [16,41]. Unlike these single or more exotic food items, beans are a familiar staple food product in cultures around the world, are of reasonable cost, and come in numerous varieties [9,18]. Pinto beans are the most widely consumed legume among US Hispanics [18].

US federal nutrition assistance programs like WIC, the Supplemental Nutrition Assistance Program (SNAP), and the Expanded Food and Nutrition Education Program (EFNEP) focus on improving the nutrition status of limited resource or otherwise at-risk adults and children to reduce disparities in food access, improve nutrition knowledge, and ultimately improve health. Knowledge and attitudes towards the perceived healthfulness of traditional or familiar foods such as beans, and the relationship of these views to the degree of acculturation are important concerns for better understanding of dietary choices and tailoring nutrition messages among low income groups [10,19,27]. Very few studies exist in the published literature regarding attitudes of limited resource populations towards beans or other foods they obtain through nutrition assistance programs [10,19,23,24]. Health professional awareness of stigma, status, perceived qualities of healthfulness, or desirability towards beans among consumers can be used to leverage positive cultural practices, proactively address myths and misconceptions, and evaluate knowledge of current nutrition recommendations [42]. Subsequent tailoring and strategizing of interventions to meet the needs of specific groups can improve services and health [10,23,39].

Although Hispanics comprise only 16% of the US national population, they represented almost 30% of Arizona residents in the 2011 American Community Survey [43]. Over 13% of all families in Maricopa County were considered living in poverty in 2011, but for Hispanic families this rate was almost 28% [29]. The percentage of female household heads with children under 5 years was about 50% in general, but 61% for Hispanic women [43]. These service needs are reflected in the high percentage of Hispanic women using WIC (66%) [44] and EFNEP (52%) in Maricopa County for 2011 [29].

While some survey participants were in agreement that bean consumption can lower cholesterol, blood glucose, and reduce some cancer risks, over 50% of the women reported ‘neutral’ for these statements [2–6]. It is possible that a neutral response represents indifference to the disease condition, to eating beans, or both, or that the participant did not know. Increasing awareness of these health benefits could help retain beans in the diets of limited resource groups, including immigrants such as Hispanics as they experience acculturation [10,27,33]. Promotion of beans for improving diet quality, increasing fiber intakes, reducing cholesterol, and normalizing blood glucose are important health education messages for all populations, but especially for those who are at high risk for nutritional deficits and for developing chronic diseases such as limited resource audiences [18,33].

The hypothesis that a participant’s bean knowledge would be lower among less acculturated participants was confirmed. However, the less acculturated women also were less likely to practice healthy food behaviors and safe food practices. The predictive variables of the food behavior scale, increased age, and greater acculturation suggest that women who knew more about the health benefits of beans may have had more exposure to messaging in English language media over time. However, it is important to note that regardless of acculturation level, the majority of the limited resource women expressed positive attitudes towards beans, particularly about their nutrition value and satiety qualities.

A central strength of the project was the identification of preexisting beliefs which can be used to leverage positive cultural practices, and to proactively address gaps in knowledge [36,39]. The role of beans as a vegetable that is a good source of nutrients, high in fiber, fat-free, satiating, as well as a familiar and culturally important traditional food should be emphasized in nutrition education curricula [8,9,18]. The examination of attitudes toward food is critical to grasp the cultural meaning and significance of their use, to develop strategies to increase consumption, and to evaluate if current nutrition guidelines are known [19,33]. This information gives context to recommendations and allows for the tailoring of interventions to meet the needs of priority groups [19,22–24].

The results also confirm the importance of looking at acculturation and ethnic affiliation through multiple measures as utilized by the BAS [30,35]. Relying solely on the US Census Hispanic identifier question or usage of Spanish language alone would have masked important differences and nuances among the responses of the women in the study [19,30,45].

There are some limitations to the study. These data are drawn from a cross-sectional convenience sample of predominately Mexican origin and White women who were participants at EFNEP, WIC, or an unemployment center in one major metropolitan area. These results may not be applicable to Hispanics overall, or other limited resource women, men, or women who are not involved with a nutrition assistance or job placement program. While efforts were made to pilot test the survey instrument for comprehension among a similar population, and a bicultural staff person was available to give assistance, it is possible that respondents did not understand the written questions in the self-administered instrument.

## Conclusions

Increasing awareness of the health benefits of beans may help reduce disease risk for conditions that disproportionately affect limited resource women and Hispanics. Nutrition education for disease prevention should promote bean consumption as part of a healthy lifestyle that fits with cultural traditions among Latino groups. Promotion of cultural practices may make recommendations more salient and achievable for immigrants, especially those with limited resources. Culturally appropriate health messages are essential and cannot be derived from national survey data alone. Our findings aid in the development of culturally tailored messages for the retention or increase of bean consumption in traditional and mainstream diets for disease prevention among limited resource Hispanic women living in the southwest US. These lessons can also be translated to other settings to improve health.

## Supporting Information

S1 File (SAV)
